# Identification of *Astrotactin2* as a Genetic Modifier That Regulates the Global Orientation of Mammalian Hair Follicles

**DOI:** 10.1371/journal.pgen.1005532

**Published:** 2015-09-29

**Authors:** Hao Chang, Hugh Cahill, Philip M. Smallwood, Yanshu Wang, Jeremy Nathans

**Affiliations:** 1 Department of Molecular Biology and Genetics, Johns Hopkins University School of Medicine, Baltimore, Maryland, United States of America; 2 Howard Hughes Medical Institute, Johns Hopkins University School of Medicine, Baltimore, Maryland, United States of America; 3 Department of Neuroscience, Johns Hopkins University School of Medicine, Baltimore, Maryland, United States of America; 4 Department of Ophthalmology, Johns Hopkins University School of Medicine, Baltimore, Maryland, United States of America; Stanford University School of Medicine, UNITED STATES

## Abstract

Planar cell polarity (PCP) signaling controls the global orientation of surface structures, such as hairs and bristles, in both vertebrates and invertebrates. In *Frizzled6* ^*-/-*^ (*Fz6* ^*-/-*^) mice, hair follicle orientations on the head and back are nearly random at birth, but reorient during early postnatal development to eventually generate a nearly parallel anterior-to-posterior array. We report the identification of a naturally occurring exon 5 deletion in *Astrotactin2* (*Astn2*) that acts as a recessive genetic modifier of the *Fz6* ^*-/-*^ hair patterning phenotype. A genetically engineered *Astn2* exon 5 deletion recapitulates the modifier phenotype. In *Fz6* ^*-/-*^ *;Astn2* ^*ex5del/del*^ mice, hair orientation on the back is subtly biased from posterior-to-anterior, leading to a 180-degree orientation reversal in mature mice. These experiments suggest that Astn2, an endosomal membrane protein, modulates PCP signaling.

## Author Summary

Hair, feather, and scale patterns are a universal feature of vertebrate surface morphology. These patterns are under precise genetic control as seen by their species-specificity and by their alterations in different breeds of domesticated animals. The first clues to the mechanism of hair patterning in mammals came from genetic analyses of proteins that are homologous to a small set of Drosophila proteins that control patterning of bristles and hairs on the insect body surface and wings. The patterning process, referred to as planar cell polarity, involves a cell surface protein, Frizzled6, which is produced in skin and hair follicles. Following a chance observation that some *Frizzled6* mutant mice exhibit an unusual hair pattern in which all of the hair follicles on the posterior half of the back have reversed orientations, we have identified a single spontaneous mutation that accounts for this reversal. The mutation removes a single coding exon from the gene coding for the membrane protein Astrotactin2. Interestingly, a closely related protein, Astrotactin1, has been implicated in directed neuronal migration along a glial substrate, suggesting a mechanistic connection between patterning mechanisms in skin and brain.

## Introduction

In complex multi-cellular organisms, individual surface structures such as hairs, feathers, scales, and bristles typically exhibit a high degree of spatial order. In birds and mammals, the stereotyped orientations of feathers and hairs reflect the underlying orientations of follicles within the dermis. Hair follicle orientation is controlled by planar cell polarity (PCP) signaling, as determined by the changes in follicle orientation associated with mutations in the core PCP genes *Frizzled6* (*Fz6*), *Celsr1*, and *Van Gogh-like2* (*Vangl2*) in mice [1–5]. In the absence of *Fz6*, the initial orientations of hair follicles on the head and back appear to be largely randomized, in contrast to the nearly parallel orientations of follicles on most of the body surface of wild type (*WT*) mice.

During the first postnatal week, hair follicles in *WT* mice undergo a subtle reorientation, referred to as “refinement”, which minimizes angular differences among neighboring follicles. This process also leads to a more precise alignment of follicles with the body axes (on the back) or with local anatomic structures (on the limbs) [2,3]. In *Fz6* ^*-/-*^ mice, the refinement process is associated with far larger angular reorientations than in *WT* mice, presumably because *Fz6* ^*-/-*^ follicles exhibit a greater diversity of initial orientations [2,3]. In *Fz6* ^*-/-*^ back skin, this process leads initially to a series of large-scale patterns, such as whorls, most of which disappear by postnatal day (P)10-P15 as the field of follicle vectors progressively aligns along an anterior-to-posterior direction.

Current evidence suggests that PCP proteins are essential for cell-to-cell propagation and intracellular interpretation of polarity information, but the molecules and mechanisms responsible for setting up the initial asymmetry in spatial information remain unknown [6]. In the present study, we identify a genetic modifier of the PCP hair patterning phenotype that imposes a large-scale asymmetry on hair follicle orientation.

## Results and Discussion

**Fig 1:**
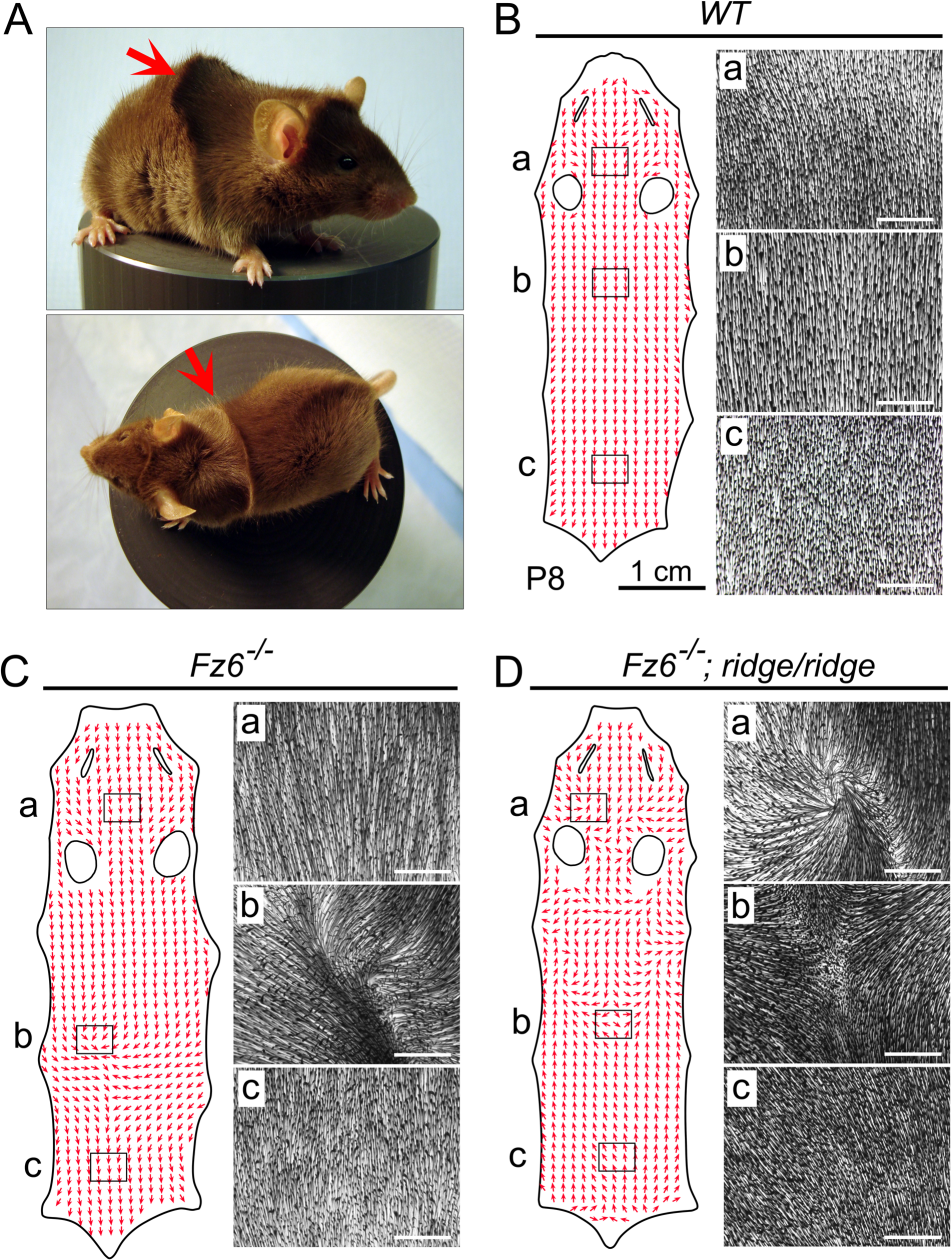
The *ridge* phenotype. (A) Side and top views of a *Fz6* ^*-/-*^ *;ridge/ridge* mouse at approximately one month of age. Arrows indicate the single transverse ridge hair pattern on the back. (B-D) Hair orientation (red arrows) on flat-mounted back skins from WT, *Fz6* ^*-/-*^, and *Fz6* ^*-/-*^ *;ridge/ridge* mice at P8. Images to the right of each flat mount correspond to the boxed regions labeled a-c and illustrate the correlation between vector scoring (red arrows) and the raw data (montage images showing follicle orientations). Rostral is at the top; caudal is at the bottom. The narrow slits and oval holes correspond to the locations of the eyes and ears, respectively. WT follicles are almost perfectly aligned in an anterior-to-posterior direction (B). Most *Fz6* ^*-/-*^ follicles are aligned in an anterior-to-posterior direction, except for a region in the mid-back (C). *Fz6* ^*-/-*^ *;ridge/ridge* follicles in the caudal half of the back exhibit a uniformly reversed (i.e. posterior-to-anterior) orientation (D). White scale bars, 1 mm.

This work began with the chance discovery of an unusual and stereotyped hair pattern among siblings in a *Fz6* ^*-/-*^ intercross, referred to hereafter as the *ridge* phenotype. This phenotype is characterized by a transverse ridge across the back, which arises when hairs in the upper back that are oriented in an anterior-to-posterior direction encounter hairs on the lower back that are oriented in a posterior-to-anterior direction (Figs 1 and S1). The *ridge* pattern is not observed in typical *Fz6* ^*-/-*^ mice. As seen in Figs 1 and S1, typical *Fz6* ^*-/-*^ back skins at P8 exhibit limited deviations from the strictly anterior-to-posterior follicle orientation of *WT* follicles.

**Fig 2:**
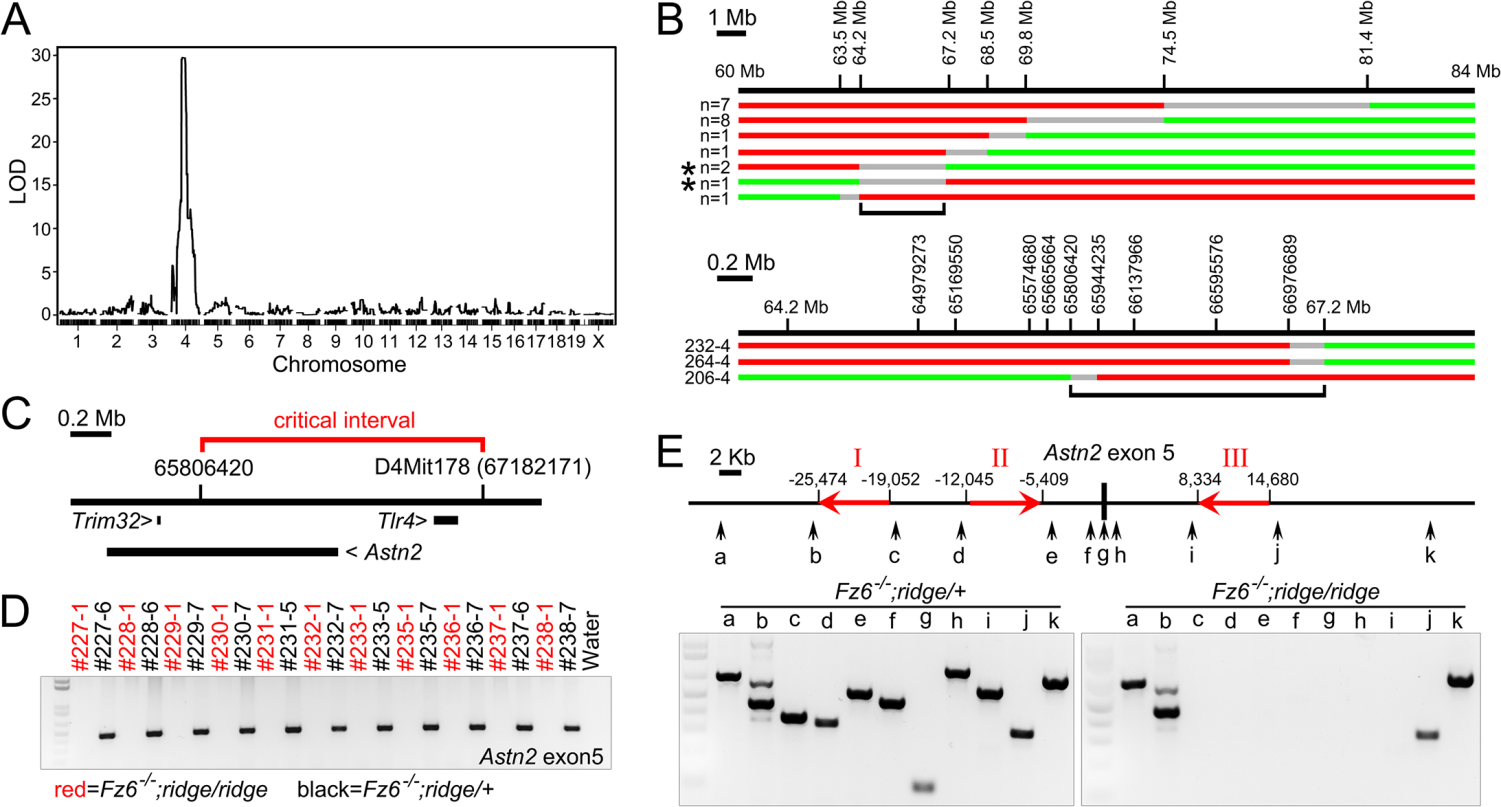
Identification of *Astn2* as the *ridge* gene. (A) Whole genome multipoint LOD score for the *ridge* locus based on SNP typing of 1,449 loci. (B) Recombination mapping of the critical interval. The locations of microsatellite markers on chromosome 4 are shown above the maps of the recombinant *ridge* chromosomes, with the number of independent chromosomes of each type indicated at left. Red, region derived from the *ridge* chromosome; green, region derived from the WT chromosome; grey, region encompassing the cross-over point. Low-resolution (top) and high-resolution (bottom) maps. For the three recombinant chromosomes shown at the bottom (and indicated by asterisks in the upper part of the figure), the *ridge* phenotype was confirmed by examining at least nine progeny from each mouse that inherited the original recombination event. Black brackets demarcate the critical interval. (C) Locations of the three genes within or adjacent to the critical interval. (D) PCR amplification of *Astn2* exon 5 from *Fz6* ^*-/-*^ *;ridge/ridge* and *Fz6* ^*-/-*^ *;ridge/+* siblings (each 3-digit number indicates a different sibship). All *Fz6* ^*-/-*^ *;ridge/+* samples give the expected WT PCR product and all *Fz6* ^*-/-*^ *;ridge/ridge* samples give no PCR product. (E) PCR reactions in the neighborhood of *Astn2* exon 5 (locations shown by vertical arrows) show that the *ridge* allele is missing ~30 kb, consistent with an homologous recombination event between the LINE elements designated ‘I’ and ‘III’ (red arrows show location and 5’ to 3’ orientation).

Additional crosses established that the *ridge* phenotype segregates as a recessive trait and is only observed in the absence of *Fz6*. As the genetic background of our *Fz6* ^*-/-*^ line consisted of contributions from C57Bl6/J and SV129, as well as an indeterminate contribution from a Flp-expressing line, we guessed that the *Fz6* ^*-/-*^ line might harbor sufficient genetic diversity that a genome-wide SNP screen could identify the locus responsible for the *ridge* phenotype. This strategy revealed a single linkage peak based on typing of 1,449 loci across the genome in 43 *ridge+* and 39 *ridge-* progeny from a *Fz6* ^*-/-*^ intercross that was segregating the *ridge* phenotype. The peak resides on chromosome 4 and has a multipoint LOD score of 30 (Fig 2A).

To narrow the region within which the *ridge* locus resides, we scored hair patterns and polymorphic markers flanking the critical interval in >1,500 progeny of *Fz6* ^*-/-*^ *;ridge/ridge* x *Fz6* ^*-/-*^ *;ridge/+* parents, and then fine-mapped the recombination breakpoints in the subset of progeny that exhibited a recombination event within the critical interval (Fig 2B). This analysis narrowed the critical interval to a 2.3 Mb segment encompassing or adjacent to the genes for *Toll-like receptor4* (*Tlr4*), *Astrotactin2* (*Astn2*), and *Trim32*, a gene embedded within intron 16 of the *Astn2* gene (Fig 2C). A 74.7 kb spontaneous deletion that overlaps the *Tlr4* gene, and that eliminates *Tlr4* mRNA and protein production [7,8], was crossed into the *Fz6* ^*-/-*^ line and found to have no effect on hair patterning, indicating that the *ridge* phenotype does not arise from a loss of *Tlr4* function. PCR amplification and sequencing of all of the exons of these three genes showed only one difference between control C57Bl6/J and *ridge* chromosomes: a consistent failure to amplify *Astn2* exon 5 from *ridge* chromosomes (Figs 2D and S2). Tests with ten additional PCR primer pairs in the flanking introns revealed a ~30 kb deletion that encompasses *Astn2* exon 5 and has endpoints within a pair of LINE elements (Fig 2E).

**Fig 3:**
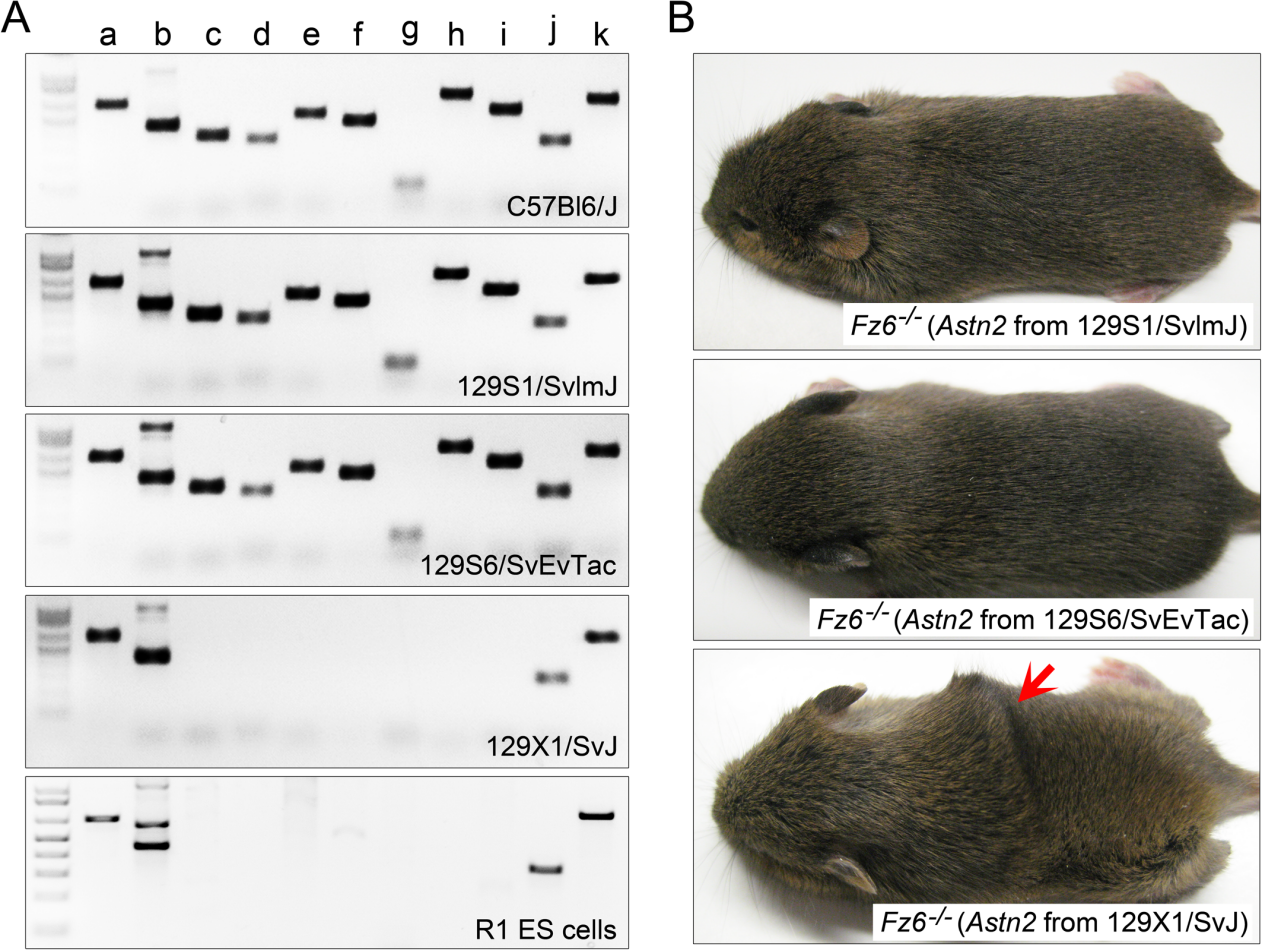
Origin of the *Astn2* exon 5 deletion. (A) PCR analysis of genomic DNA in the neighborhood of *Astn2* exon 5, as shown in Fig 2E. The ~30 kb deletion is present in 129X1/SvJ mice and R1 ES cells, but is absent from C57Bl6/J, 129S1/SvlmJ, and 129S6/SvEvTac mice. (B) The *Astn2* locus from the three 129 mouse lines shown in (A) was bred to homozygosity in a *Fz6* ^*-/-*^ background. The *ridge* phenotype was observed only in the presence of the 129X1/SvJ *Astn2* locus; mice were photographed at P14.

To search for the origin of the *Astn2* exon 5 deletion, we analyzed the ES cells that were used to generate the targeted *Fz6* null allele [1]. This ES cell line (“R1”) was derived by Nagy et al [9] from a cross between two 129/Sv lines [10–12]. PCR typing showed that the ~30 kb deletion is present in both R1 ES cells and in 129X1/SvJ mice, but not in the closely related 129S1/SvlmJ or 129S6/SvEvTac lines (Fig 3A). NextGen sequencing of genomic DNA from the critical interval confirmed the presence of this deletion in 129X1/SvJ and *Fz6* ^*-/-*^ *;ridge/ridge* lines but not in the 129S1/SvlmJ or 129S6/SvEvTac lines (S3 Fig). Importantly, when the *Astn2* allele present in each of the three 129 lines was crossed into the *Fz6* ^*-/-*^ background and assessed in the homozygous state, only the 129X1/SvJ-derived *Astn2* allele produced the *ridge* phenotype (Fig 3B).

**Fig 4:**
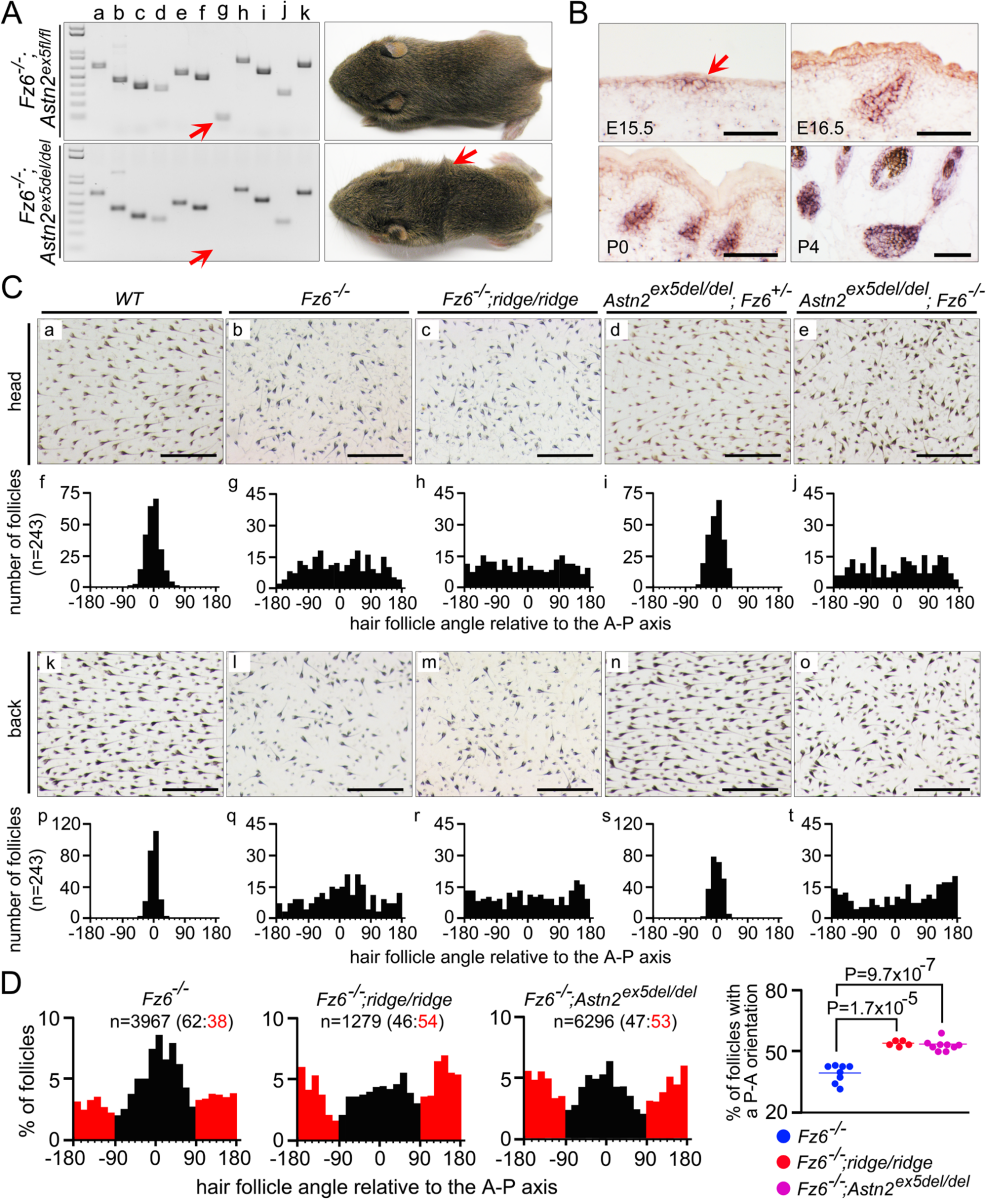
Targeted deletion of *Astn2* exon 5 and quantitative analysis of hair follicle orientations in early postnatal back skins. (A) PCR analysis of genomic DNA in the neighborhood of *Astn2* exon 5 (left) and gross appearance of P14 mice (right). Red arrow indicates the exon 5 PCR product. *Fz6* ^*-/-*^ *;Astn2* ^*ex5fl/fl*^ mice (with an intact *Astn2* exon 5) lack a ridge, whereas *Fz6* ^*-/-*^ *;Astn2* ^*ex5del/del*^ mice (lacking *Astn2* exon 5) have a ridge. (B) By in situ hybridization, *Astn2* is expressed in hair follicles beginning at the placode stage (E15.5; arrow) and continuing throughout the period of follicle maturation. Scale bars, 0.1 mm. (C) Flat mount head and lower back skin of the indicated genotypes at P3. Quantifications of follicle angles are shown for each genotype beneath the flat mount images (n = 3 mice per genotype). For each skin, 81 follicle angles were determined for a set of follicles closest to the grid points on a 9 x 9 grid (see Methods for further details). Zero degrees corresponds to anterior-to-posterior; 180 and -180 degrees corresponds to posterior-to-anterior. Scale bars, 0.5 mm. *Astn2* exon 5 deletion has no effect on follicle orientation in a *Fz6* ^*+/-*^ background. *Fz6* ^*-/-*^ *;Astn2* ^*ex5del/del*^ is indistinguishable from *Fz6* ^*-/-*^ *;ridge/ridge*. (D) Quantification of follicle orientations on the lower backs of eight *Fz6* ^*-/-*^, five *Fz6* ^*-/-*^ *;ridge/ridge*, and nine *Fz6* ^*-/-*^ *;Astn2* ^*ex5del/del*^ mice at P3. Left, histograms shows all of the follicles quantified per genotype (n). Follicles with an anterior-to-posterior direction are shown in black; follicles with a posterior-to-anterior direction are shown in red. The ratio of the two classes is indicated above each histogram. Right, scatter plot showing the percent of follicles with a reversed (i.e. posterior-to-anterior) orientation for each skin. P-value was calculated with a student’s t-test.

The data presented thus far provide strong correlative evidence that the *Astn2* exon 5 deletion causes the *ridge* phenotype. To definitively test this hypothesis, we used gene targeting in ES cells derived from 129S6/SvEvTac mice to generate a conditional allele in which *Astn2* exon 5 is flanked by *loxP* sites (S4 Fig). When this conditional allele is made homozygous in a *Fz6* ^*-/-*^ background (*Fz6* ^*-/-*^ *;Astn2* ^*ex5fl/fl*^) the hair pattern is indistinguishable from that seen in *Fz6* ^*-/-*^ mice, i.e. it lacks a ridge. By contrast, *Fz6* ^*-/-*^ *;Astn2* ^*ex5del/del*^ mice, which lack *Astn2* exon 5 (following Cre-mediated germ-line recombination of the *Astn2* ^*ex5fl*^ allele), show a *ridge* phenotype indistinguishable from that seen in the original *Fz6* ^*-/-*^ *;ridge/ridge* mice (Fig 4A). As with the naturally occurring *ridge* genotype, the *Astn2* ^*ex5del/del*^ genotype does not produce a hair patterning phenotype on a *Fz6* ^*+/-*^ background. These experiments demonstrate that a small deletion encompassing *Astn2* exon 5 is responsible for the *ridge* phenotype, thereby identifying a mammalian modifier locus and revealing its origin as a recent spontaneous deletion.

It is possible that the naturally occurring ~30 kb *ridge* deletion eliminates transcriptional regulatory sequences in addition to eliminating *Astn2* exon 5. Such a possibility is less likely for the engineered exon 5 deletion (*Astn2* ^*ex5del*^), which is only 1.07 kb in length. The transcription start sites of the *Trim32* and *Astn2* genes are at distances of 443 kb and 345 kb from *Astn2* exon 5 (Fig 2C). RT-PCR analysis of *Trim32* transcripts in embryonic day (E)15.5 *Fz6* ^*-/-*^ and *Fz6* ^*-/-*^ *;ridge/ridge* skin showed qualitatively similar expression levels (S5 Fig). A similar RT-PCR analysis of *Astn2* transcripts in E15.5 *Fz6* ^*-/-*^, *Fz6* ^*-/-*^ *;ridge/ridge*, and *WT* skin also showed qualitatively similar expression levels (S6A and S6B Fig). Although we cannot exclude the formal possibility that sequences in or immediately adjacent to *Astn2* exon 5 regulate the expression of a more distant gene and that the *ridge* phenotype reflects perturbations in that regulation, the weight of the evidence supports the conclusion that the *ridge* phenotype reflects the absence of *Astn2* exon 5 coding sequences.

Astn2 and its close homologue Astn1 have been implicated in neuronal migration along glial scaffolds [13,14]. Astn1 and Astn2 are predicted to have a signal peptide, two transmembrane domains, and an unusual transmembrane topography in which both N- and C-termini reside on the extracellular face of the membrane (S6C and S6D Fig). Both proteins localize to endosomes, and are expressed in multiple tissues during development [14]. By in situ hybridization, we observed *Astn2* expression in hair follicles starting at the earliest stage of their development (Fig 4B). Although the precise mechanism of action of the Astrotactins is still unclear, their endosomal localization suggests that they might be involved in recycling of plasma membrane proteins [14]. Interestingly, Devenport et al. [15] observed that PCP protein complexes in the developing epidermis are internalized into endosomes and then reassembled at the plasma membrane with every cell division. These observations suggest the possibility that an alteration in Astn2 might modify the *Fz6* ^*-/-*^ phenotype by affecting PCP protein trafficking. If correct, this hypothesis would also imply that *Fz6* ^*-/-*^ embryos retain some level of PCP signaling in the skin.

Deletion of *Astn2* exon 5 leads to an in-frame deletion of 36 amino acids in the predicted cytosolic domain, a region with no homology to any proteins other than Astn1. Interestingly, constitutive alternative splicing leads to frequent skipping of *Astn2* exon 4, which leads to an in-frame deletion of 52 amino acids, also in the predicted cytosolic domain (Figs S6 and S7). Constitutive exon 4 skipping implies that large changes in the putative intracellular domain are compatible with protein stability and function, which suggests that deletion of exon 5 may alter but not abolish Astn2 function.

How might deletion of *Astn2* exon 5 influence hair follicle development to uniformly reverse the orientations of thousands of follicles in *Fz6* ^*-/-*^ *;ridge/ridge* mice? The answer to this question could be related to the striking changes in orientation that occurs among *Fz6* ^*-/-*^ follicles during early postnatal development. As noted in the Introduction, at birth, *Fz6* ^*-/-*^ mice show hair follicle orientations on the back that appear to be approximately random, but over the first 1–2 postnatal weeks, these follicles reorient to generate a series of increasingly organized macroscopic patterns, eventually reorienting in an anterior-to-posterior direction. Our earlier work suggested that the reorientation process obeys a local consensus rule that minimizes angular differences among neighboring follicles [2,3]. Computer simulations demonstrated that this process efficiently enhances the amplitude of any global bias in initial orientation while simultaneously suppressing random orientation noise, with the result that a small initial orientation bias produces a uniform reorientation of all follicles along the direction defined by that bias [2].

An initial clue to the mechanism of follicle orientation reversal in *Fz6* ^*-/-*^ *;ridge/ridge* mice emerged when we quantified hair follicle orientations on the head and lower back of *Fz6* ^*-/-*^, *Fz6* ^*-/-*^ *;ridge/ridge*, and *Fz6* ^*-/-*^ *;Astn2* ^*ex5del/del*^ mice at P3 (Fig 4C). On the lower back, *Fz6* ^*-/-*^ *;ridge/ridge* and *Fz6* ^*-/-*^ *;Astn2* ^*ex5del/del*^ follicles show a subtle posterior-to-anterior bias, whereas *Fz6* ^*-/-*^ follicles show a subtle anterior-to-posterior bias (compare panels q vs. r and t in Fig 4C). This trend was less apparent on the head (compare panels g vs. h and j in Fig 4C). To extend this analysis, we quantified the orientations of >11,500 follicles from the lower backs of eight *Fz6* ^*-/-*^, five *Fz6* ^*-/-*^ *;ridge/ridge*, and nine *Fz6* ^*-/-*^ *;Astn2* ^*ex5del/del*^ mice at P3 (Fig 4D). The results confirm the directional bias noted above, with pairwise P-values of 1.7x10^-5^ for the *Fz6* ^*-/-*^ vs. *Fz6* ^*-/-*^ *;ridge/ridge* comparison and 9.7x10^-7^ for the *Fz6* ^*-/-*^ vs. *Fz6* ^*-/-*^ *;Astn2* ^*ex5del/del*^ comparison (student’s t-test). The P-value for the comparison of *Fz6* ^*-/-*^ vs. the combination of *Fz6* ^*-/-*^ *;ridge/ridge* and *Fz6* ^*-/-*^ *;Astn2* ^*ex5del/del*^ is 3.1x10^-9^. Interestingly, the follicle orientation histograms from all three genotypes exhibit minima at orientations perpendicular to the anterior-posterior axis (Fig 4D), suggesting an additional bias favoring follicle orientations that are either parallel or anti-parallel to this axis.

These quantitative analyses suggest that loss of *Astn2* exon 5 either (1) continuously acts to reorient follicles in the lower back in a posterior-to-anterior direction, or (2) creates an initial posterior-to-anterior orientation bias, which is subsequently enhanced by the local refinement process. Although we cannot, at present, distinguish between these alternative models, the morphologic data imply that dramatically different follicle orientation patterns in mature skin can be consistently generated from subtly different patterns in immature skin. The performance of this system is all-the-more-remarkable because, in the *Fz6* ^*-/-*^ background, the field of immature follicle vectors has a very low signal-to-noise ratio.

PCP signaling plays a central role in a wide variety of developmental processes. In addition to hair follicle orientation, these include neural tube closure, the orientation of motile cilia and of vestibular and auditory hair cells, and axon guidance [6]. The shared dependence of these processes on PCP signaling suggests that insights obtained from studying any one of them may shed light on the others. The relationship between hair follicle orientation and axon guidance is especially intriguing and is emphasized by the requirement for Celsr and Frizzled family members in both processes [5,16,17] and by the partial interchangeability of Fz6 and its close homologue Fz3, which controls axon guidance [18]. The role of Astrotactins in both neuronal migration and hair follicle orientation suggests an even closer connection between patterning mechanisms in skin and brain.

## Materials and Methods

### Ethics statement

This study was performed in strict accordance with the recommendations in the Guide for the Care and Use of Laboratory Animals of the National Institutes of Health. All of the animals were handled according to approved Institutional Animal Care and Use Committee (IACUC) protocol MO13M469 of the Johns Hopkins Medical Institutions.

### Mouse lines

*Fz6* ^*-/-*^ mice are described in Guo et al. [1]. 129X1/SvJ, 129S1/SvlmJ and the *Tlr4* deletion (JAX #003752) lines were purchased from Jackson Laboratories. The 129S6/SvEvTac line was purchased from Taconic.

### Production of *Astn2* ^*ex5fl/fl*^ mice

The *Astn2* floxed exon5 targeting construct was electroporated into MC1 ES cells (from 129S6/SvEvTac-mice; a kind gift from Mitra Cowan) and plated in G418 and ganciclovir for positive and negative selection. Colonies were screened by Southern blotting, and clones carrying the targeted allele were injected into C57BL/6 blastocysts. Chimeras were bred to C57BL/6, and the *FRT*-flanked *PGK-neo* cassette was removed by crossing to germline *Flp* mice to generate the *Astn2* ^*ex5fl*^ allele. The *Astn2* ^*ex5del*^ allele was generated by crossing mice carrying *Astn2* ^*ex5fl*^ to mice carrying germline *Sox2-Cre* [19] (*Tg(Sox2-Cre)1Amc/J*; from Jackson Laboratories).

### Phenotyping, genotyping, and mouse husbandry

For meiotic mapping, *Fz6* ^*-/-*^;*ridge/+* and *Fz6* ^*-/-*^;*ridge/ridge* progeny of *Fz6* ^*-/-*^;*ridge/+* x *Fz6* ^*-/-*^;*ridge/ridge* parents were phenotyped at ~P8-P10 by visual inspection of the hair pattern (i.e. examined for the presence or absence of the transverse ridge), and genotyped by scoring microsatellite insertion/deletion variants with the PCR primers listed in S1 Table. High resolution mapping of the critical interval was performed by SNP genotyping with the PCR primers listed in S2 Table. PCR primers for amplifying the 23 *Astn2* exons are listed in S3 Table. PCR primers for mapping the ~30 kb deletion encompassing *Astn2* exon 5 (Fig 2E) are listed in S4 Table. RT-PCR primers for amplifying *Dbc1* and *Trim32* are listed in S5 Table.

### Genome-wide SNP screen and LOD score calculation

An Illumina mouse SNP array with 1,449 loci was used to type 43 *ridge+* and 39 *ridge-* progeny from a *Fz6* ^*-/-*^ intercross that was segregating the *ridge* phenotype. The multipoint LOD score was calculated using R software with the quantitative trait locus (QTL) bioinformatics add-on package Version 1.21–2 (release March 18, 2011; http://www.rqtl.org). The calculation used a hidden Markov model with the Haley-Knott regression. The highest LOD score was 29.7 on Chromosome 4, with the peak at position 63.65.

### Hybridization capture and NextGen sequencing

Genomic DNA from 129S1/SvlmJ, 129S6/SvEvTac, 129X1/SvJ, and *Fz6* ^*-/-*^ *;ridge/ridge* mice was purified from brain tissue by proteinase K digestion and CsCl centrifugation, fragmented to a mean size of ~350 bp, captured on a custom designed Agilent SureSelect oligonucleotide array that covered all non-repetitive sequences in the interval 6.58–6.72 Mb on chromosome 4 (mouse genome build 38), and subjected to 150 base paired-end sequencing on an Illumina MySeq to a mean coverage depth of ~50X.

### Skin flat mount analysis

Skin flat mounts were prepared as described in Chang et al [20]. To visualize follicles using the endogenous melanin pigment, the dorsal back skin (at P3 and P8) was dissected and flattened by pinning its edges to a flat Sylguard surface, fixed overnight in 4% paraformaldehyde in PBS, dehydrated through a graded alcohol series, and then clarified with benzyl benzoate:benzyl alcohol (BBBA) in a glass dish. Images were collected with a dissecting microscope.

### Quantification of follicle orientations

Hair follicle orientations were scored one at a time by placing the image of a freely rotatable vector over the skin flatmount image (3.2 mm x 2.5 mm), superimposing the vector on the follicle of interest, and assessing the best fitting vector orientation by visual inspection. Two sampling strategies were used. For low-density sampling (Fig 4C), orientations were determined only for the 81 follicles closest to each point of intersection of the nine vertical and nine horizontal lines in a 2.88 mm x 2.25 mm grid overlaid on each image. For high-density sampling (Fig 4D), all follicles within each image were scored. Vector orientations were measured in Photoshop and ImageJ. Statistical comparisons were performed in Microsoft Excel. To assess the reproducibility of the scoring method, images of two P3 back skin flat mounts (one *Fz6* ^*-/-*^ and the other *Fz6* ^*-/-*^ *;Astn2* ^*ex5del/del*^) were rotated 180 degrees, and follicle orientations for all four images (two original and two rotated; n is approximately 700 follicles per image) were determined by an individual who was blinded to the genotypes and to the relatedness of the images. As shown in S8 Fig, when corrected for the 180 degree rotation, the distributions of follicle angles in the two rotated images were found to be nearly identical to the distributions in the original images, and each image reproduced the distinctive genotype-specific patterns shown in Fig 4D, which were based on quantification of 22 back skin images.

### In situ hybridization

In situ hybridization was performed as described [21]. Digoxigenin-labeled riboprobes were transcribed using T7 RNA polymerase from the *Astn2* cDNA (coding regions within exons 19–23), which was cloned from *WT* mouse E15.5 skin by RT-PCR. Images were captured on an Imager Z1 microscope (Zeiss) using Openlab software.

## Supporting Information

S1 Fig Consistently reversed hair orientations on the lower back of *Fz6* ^*-/-*^ *;ridge/ridge* mice at P8. Hair follicle orientations in flat-mounted back skins from *Fz6* ^*-/-*^ *;ridge/ridge* (left) and conventional *Fz6* ^*-/-*^ (i.e. non-ridge) mice at P8. Rostral is at the top; caudal is at the bottom. The narrow slits and oval holes correspond to the locations of the eyes and ears, respectively. *Fz6* ^*-/-*^ *;ridge/ridge* follicles in the caudal half of the back exhibit a uniformly reversed (i.e. posterior-to-anterior) orientation. At this age, *Fz6* ^*-/-*^ follicles are predominantly aligned in an anterior-to-posterior direction, except for localized regions on the mid-back and/or head where follicles show a misalignment of ~45 degrees from the anterior-to-posterior direction.
(TIF)

S2 Fig PCR amplification of the 23 *Astn2* exons from *Fz6* ^*-/-*^ and *Fz6* ^*-/-*^ *;ridge/ridge* mice. For *Astn2* exon 5 (indicated by an asterisk), no PCR product was obtained from *Fz6* ^*-/-*^ *;ridge/ridge* mice (arrows).
(TIF)

S3 Fig NextGen sequencing of single-copy sequences captured from the 2.3 Mb critical interval using genomic DNA from 129S1/SvlmJ, 129S6/SvEvTac, 129X1/SvJ, and *Fz6* ^*-/-*^ *;ridge/ridge* mice. Histograms of the number of aligned sequencing reads are shown for a 100 kb region centered on *Astn2* exon 5. Exons 4 and 6 reside outside of this region. The three regions with no sequencing reads correspond to LINE elements that were not included in the capture array (red arrows). The map at the top shows the locations of *Astn2* exon 5 and the three LINE elements, labeled as in Fig 2E.
(TIF)

S4 Fig *Astn2* exon 5 conditional deletion strategy. (A) From top to bottom: (1) map of *WT Astn2* exon 5 region with *Bgl* I sites and Southern blot probes shown; (2) the initial gene targeted allele [*Astn2* ^*ex5fl(neo)*^] with *loxP* sites flanking exon 5 and the *Frt-Neo-Frt* (*FNF*) positive selection cassette adjacent to the 3’ *loxP* site; (3) the targeted allele after excision of the *neo* cassette by germline Flp-mediated recombination (*Astn2e* ^*ex5fl*^); and (4) the exon 5 deleted allele after germline Cre-mediated recombination (*Astn2* ^*ex5del*^). (B) Southern blot detection of the initial targeting event in 129S6/SvEvTac-derived ES cells (“MC1” ES cells). (C) PCR shows that the starting MC1 ES cells carry the intact *Astn2* exon 5 region. The PCR analysis is the same as shown in Fig 2E.
(TIF)

S5 Fig RT-PCR of *Dbc1* and *Trim32* transcripts in E15.5 skin from *Fz6* ^*-/-*^, and *Fz6* ^*-/-*^ *;ridge/ridge* embryos. For each transcript, PCR reactions were performed with the three primer pairs indicated. *Dbc1* is located ~2 Mb 5’ of the *Astn2* transcription start site. *Trim32* is located within the ~1 Mb *Astn2* transcription unit.
(TIF)

S6 Fig *WT* and *ridge Astn2* mRNA structure and predicted protein topography. (A) The 23 *Astn2* exons, showing the exon 4 skipping event, exon 5 (in red), and the locations of PCR primers used for RT-PCR. Amplification with primer pair 631/633 (shown above the map) reveals the presence or absence of exons 4 and/or 5 in mature *Astn2* transcripts. Yellow, 5’ and 3’ untranslated regions; blue, coding region. (B) RT-PCR reaction products show the presence and structure of *Astn2* transcripts from E15.5 skin from *WT*, *Fz6* ^*-/-*^, and *Fz6* ^*-/-*^ *;ridge/ridge* mice. In all three genotypes, the overall abundance of *Astn2* transcripts are similar and isoforms with and without exon 4 are present. *Astn2* transcripts in the *Fz6* ^*-/-*^ *;ridge/ridge* sample are missing exon 5. (C) Kyte-Doolittle hydropathy profile for Astn2. The locations of the predicted signal peptide and two trans-membrane segments are indicated. (D) Predicted transmembrane topography for Astn2 showing the locations of regions coded by exons 4 and 5, and regions with homology to known domains. FN III, fibronectin type III domain; MACPF, membrane attack complex/perforin domain.
(TIF)

S7 Fig Predicted amino acid sequence of mouse *Astn2*, showing the locations of exons 4 and 5. Green underline, exon 4. Purple underline, exon 5. Alternating blocks of black and blue letters represent amino acids coded within different exons. Red letters indicate locations where an intron falls within a codon. The predicted locations of the signal peptide (SP) and the two transmembrane domains (TM1 and TM2) are indicated.
(TIF)

S8 Fig Reproducibility of the follicle angle scoring methodology. Images of two P3 back skin flat mounts (one *Fz6* ^*-/-*^ and the other *Fz6* ^*-/-*^ *;Astn2* ^*ex5del/del*^) were rotated 180 degrees, and the orientations of all follicles within the four images (two original and two rotated) were determined as described in Methods. The scorer was blinded to the genotypes and to the relatedness of the images. A, B, D, E, Follicle orientation histograms are shown for the two original (A and D) and two rotated (B and E) images. For each image, the number of follicles scored and the ratio of left-to-right (black) and right-to-left (red) vectors is shown. In the original images, anterior was to the left and posterior was to the right. B’ and E’, Histograms of the rotated data set after correction for the 180-degree rotation. C and F, Distributions of follicle angles for the original images (blue lines) and the two rotated images after correction for the 180-degree rotation (red lines).
(TIF)

S1 Table PCR primers: polymorphic microsatellite insertion/deletion variants. (XLSX)

S2 Table PCR primers: SNP variants. (XLSX)

S3 Table PCR primers for amplifying Astn2 exons. (XLSX)

S4 Table PCR primers for defining the extent of the ridge deletion. (XLSX)

S5 Table RT-PCR primers for *Dbc1* and *Trim32*. (XLSX)
