# Supplementary figures and images for: Identification of Astrotactin2 as a Genetic Modifier That Regulates the Global Orientation of Mammalian Hair Follicles
Source: PLoS Genet. 2015 Sep 29;11(9):e1005532. doi: 10.1371/journal.pgen.1005532 (PMC4587951; doi:10.1371/journal.pgen.1005532)

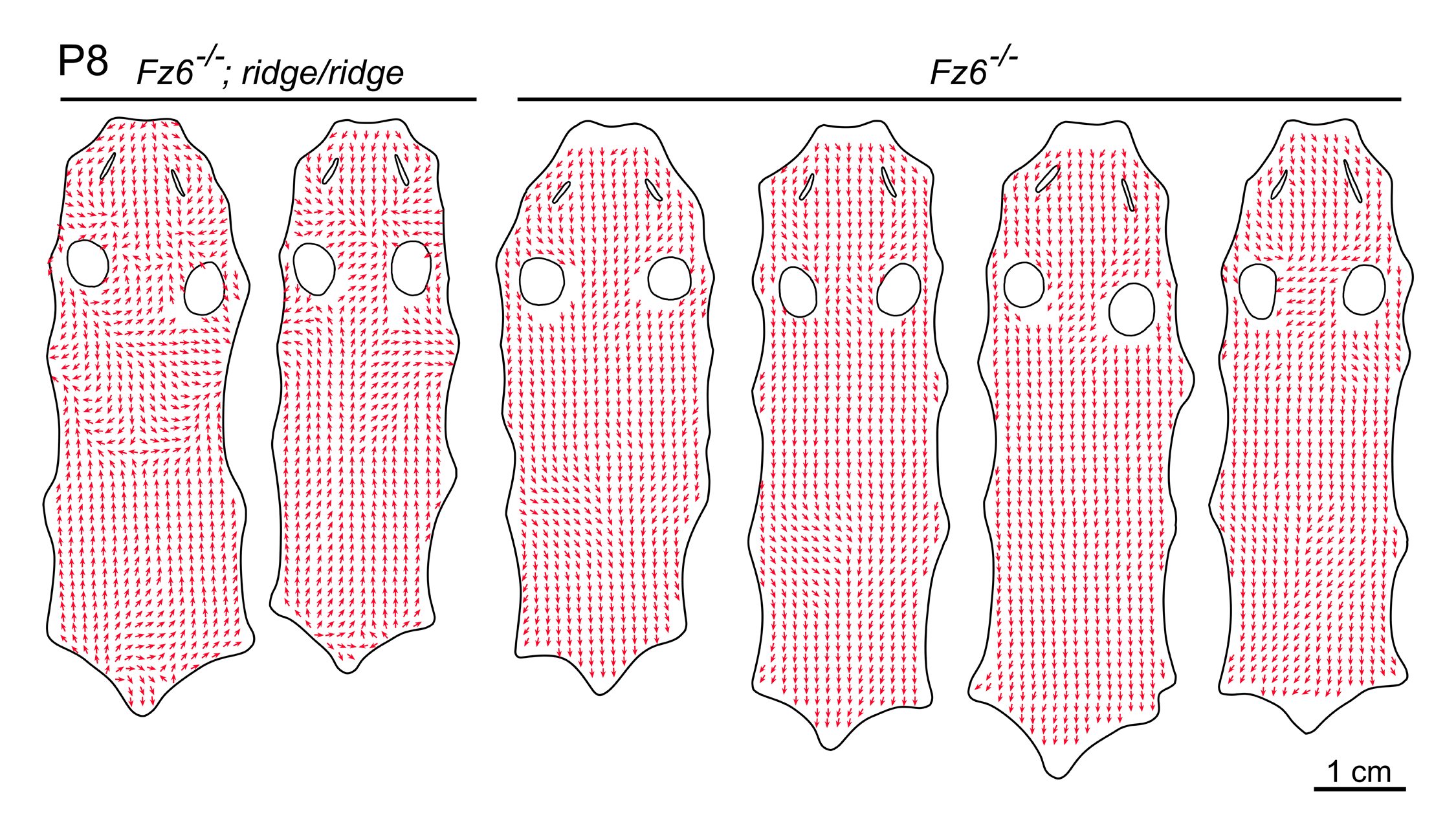

Supplement: S1 Fig — Hair follicle orientations in flat-mounted back skins from Fz6 -/- ;ridge/ridge (left) and conventional Fz6 -/- (i.e. non-ridge) mice at P8. Rostral is at the top; caudal is at the bottom. The narrow slits and oval holes correspond to the locations of the eyes and ears, respectively. Fz6 -/- ;ridge/ridge follicles in the caudal half of the back exhibit a uniformly reversed (i.e. posterior-to-anterior) orientation. At this age, Fz6 -/- follicles are predominantly aligned in an anterior-to-posterior direction, except for localized regions on the mid-back and/or head where follicles show a misalignment of ~45 degrees from the anterior-to-posterior direction. (TIF) [file pgen.1005532.s001.tif]

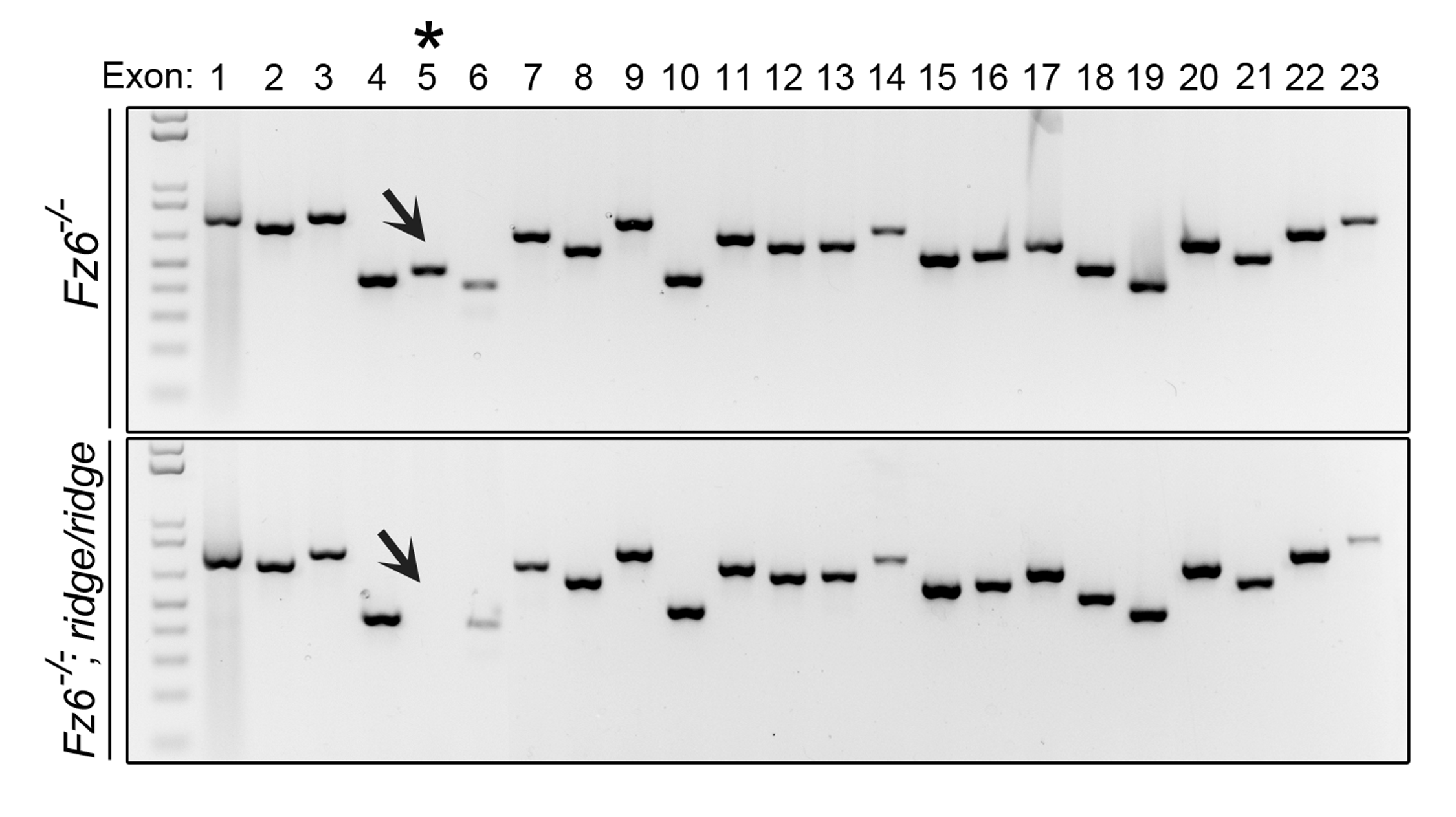

Supplement: S2 Fig — For Astn2 exon 5 (indicated by an asterisk), no PCR product was obtained from Fz6 -/- ;ridge/ridge mice (arrows). (TIF) [file pgen.1005532.s002.tif]

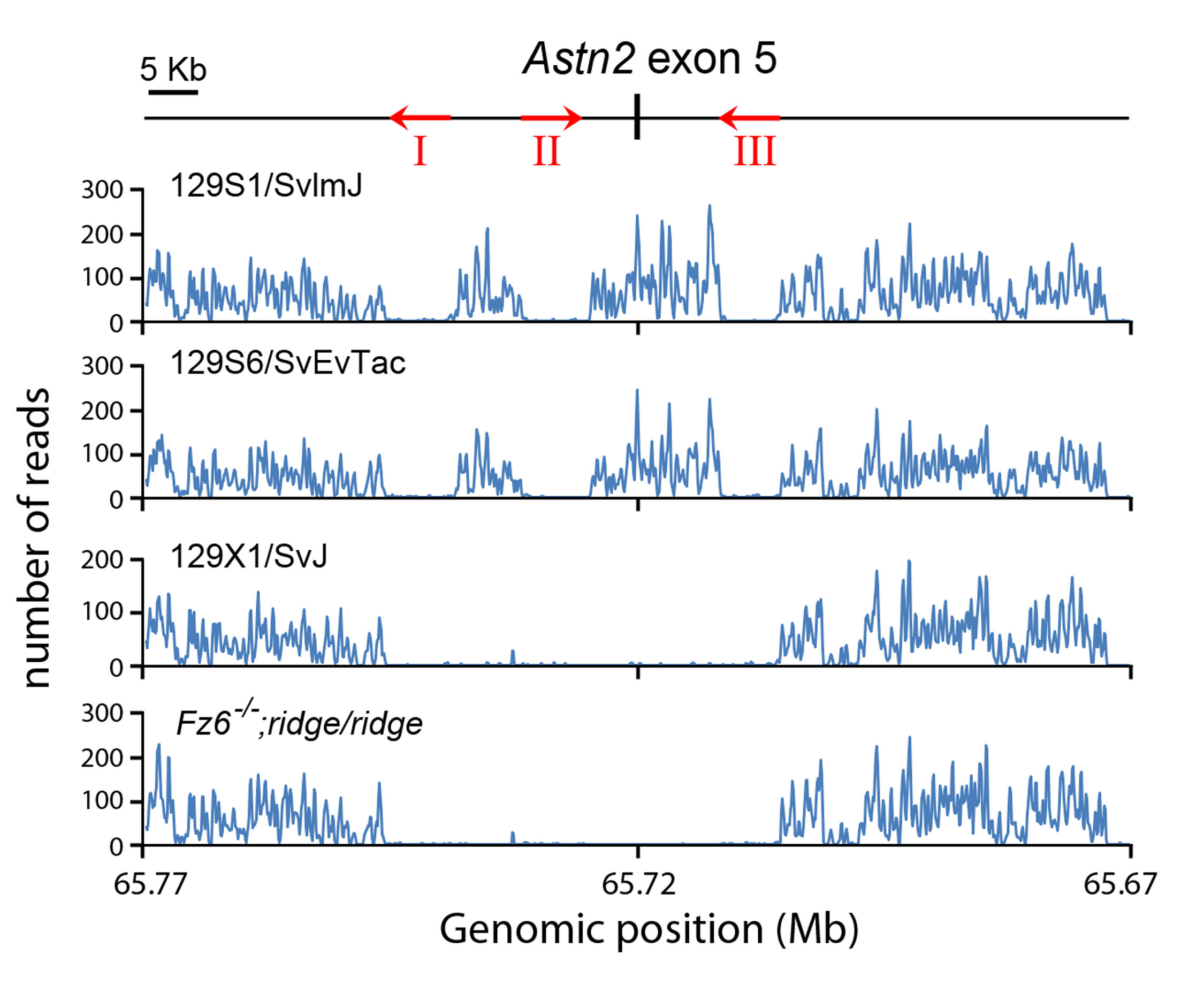

Supplement: S3 Fig — Histograms of the number of aligned sequencing reads are shown for a 100 kb region centered on Astn2 exon 5. Exons 4 and 6 reside outside of this region. The three regions with no sequencing reads correspond to LINE elements that were not included in the capture array (red arrows). The map at the top shows the locations of Astn2 exon 5 and the three LINE elements, labeled as in Fig 2E. (TIF) [file pgen.1005532.s003.tif]

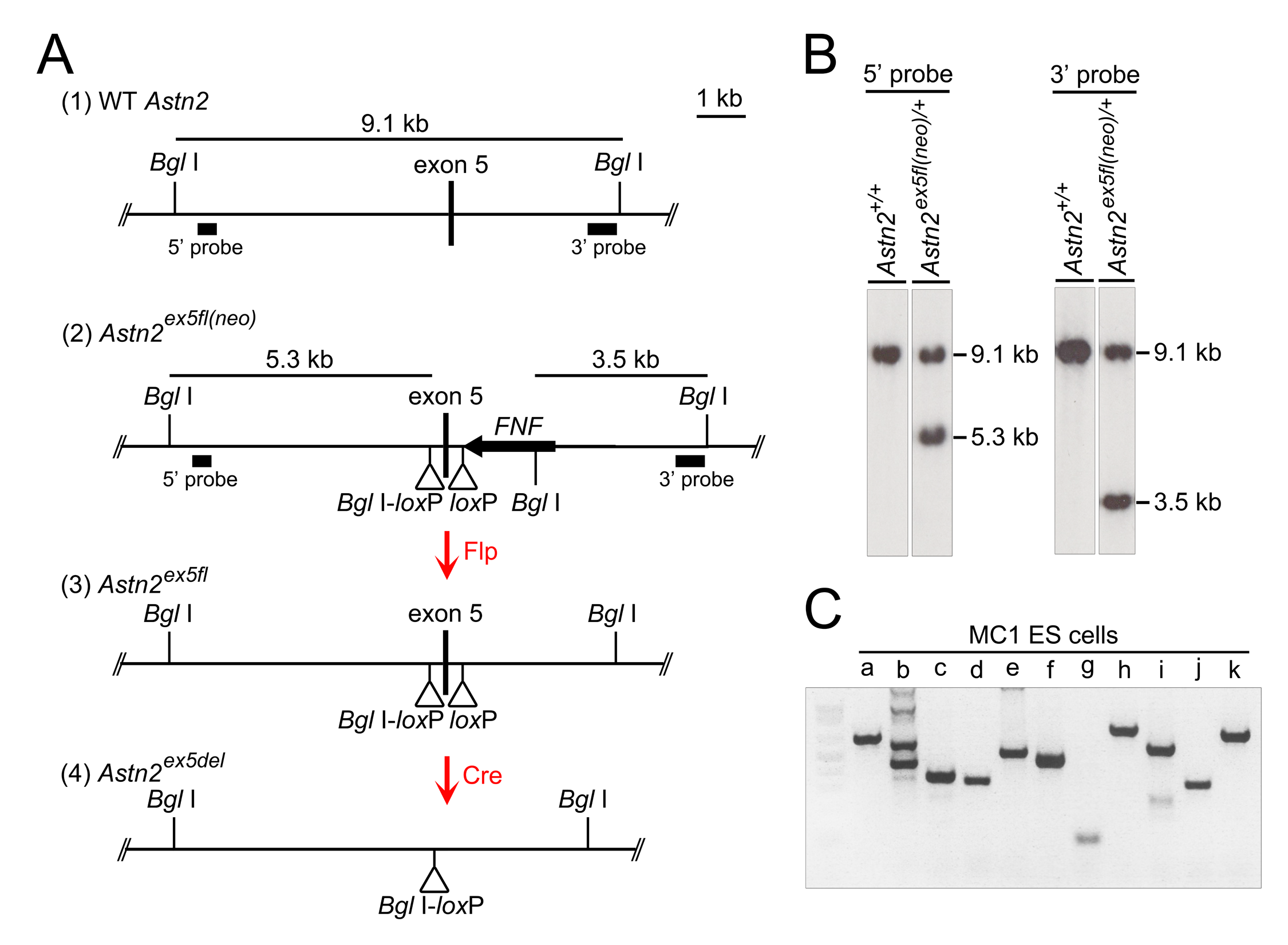

Supplement: S4 Fig — (A) From top to bottom: (1) map of WT Astn2 exon 5 region with Bgl I sites and Southern blot probes shown; (2) the initial gene targeted allele [Astn2 ex5fl(neo)] with loxP sites flanking exon 5 and the Frt-Neo-Frt (FNF) positive selection cassette adjacent to the 3’ loxP site; (3) the targeted allele after excision of the neo cassette by germline Flp-mediated recombination (Astn2e ex5fl); and (4) the exon 5 deleted allele after germline Cre-mediated recombination (Astn2 ex5del). (B) Southern blot detection of the initial targeting event in 129S6/SvEvTac-derived ES cells (“MC1” ES cells). (C) PCR shows that the starting MC1 ES cells carry the intact Astn2 exon 5 region. The PCR analysis is the same as shown in Fig 2E. (TIF) [file pgen.1005532.s004.tif]

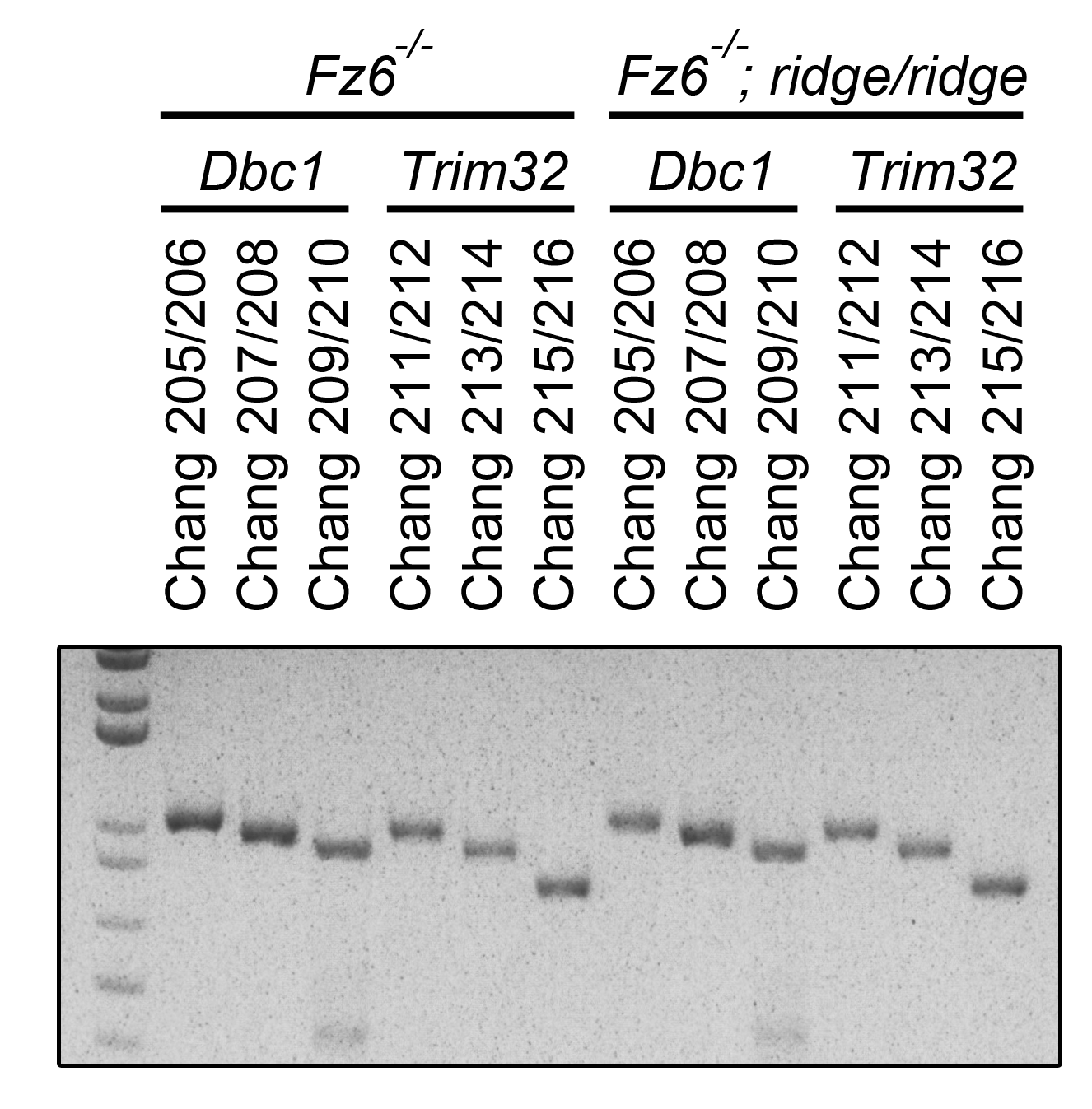

Supplement: S5 Fig — For each transcript, PCR reactions were performed with the three primer pairs indicated. Dbc1 is located ~2 Mb 5’ of the Astn2 transcription start site. Trim32 is located within the ~1 Mb Astn2 transcription unit. (TIF) [file pgen.1005532.s005.tif]

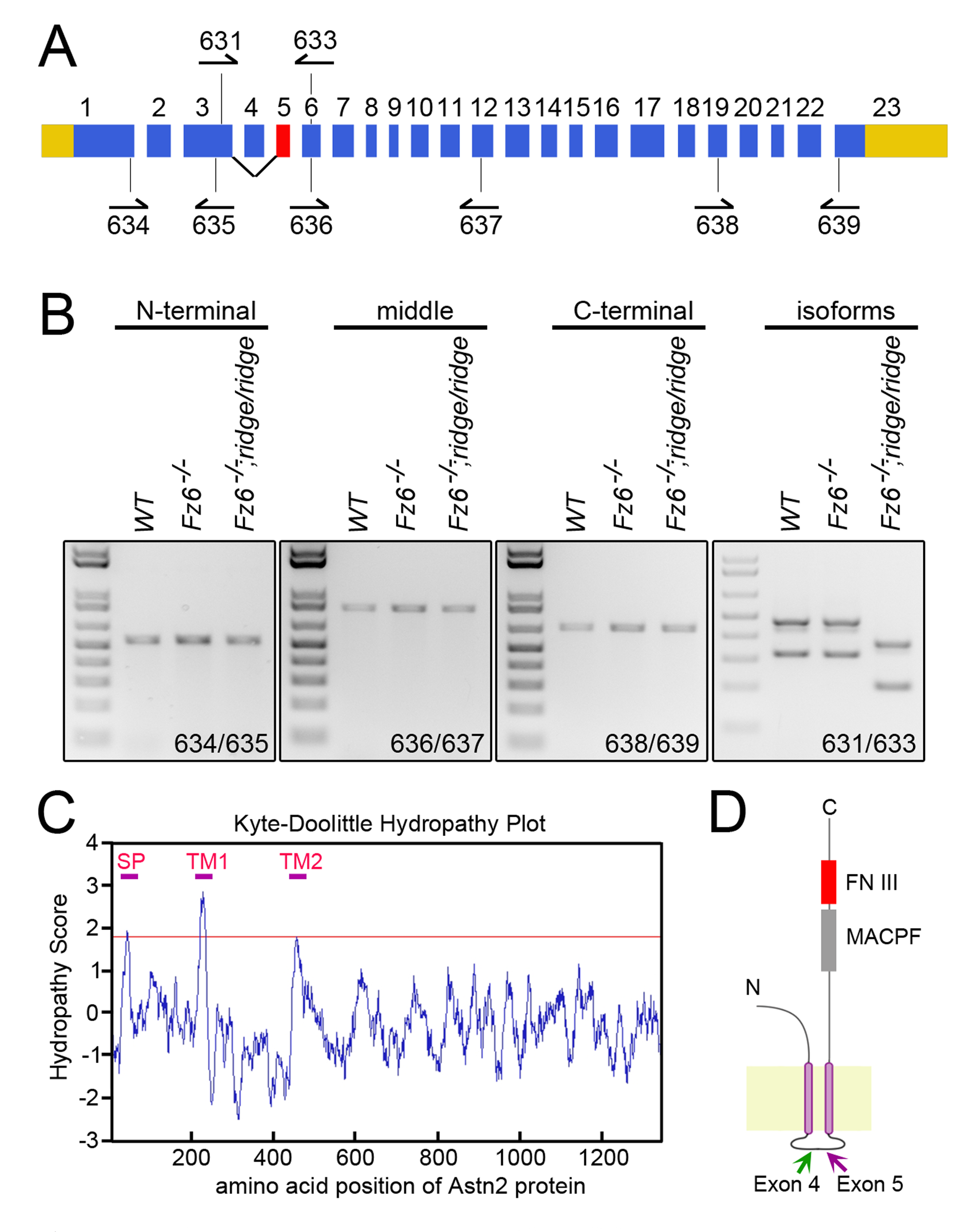

Supplement: S6 Fig — (A) The 23 Astn2 exons, showing the exon 4 skipping event, exon 5 (in red), and the locations of PCR primers used for RT-PCR. Amplification with primer pair 631/633 (shown above the map) reveals the presence or absence of exons 4 and/or 5 in mature Astn2 transcripts. Yellow, 5’ and 3’ untranslated regions; blue, coding region. (B) RT-PCR reaction products show the presence and structure of Astn2 transcripts from E15.5 skin from WT, Fz6 -/-, and Fz6 -/- ;ridge/ridge mice. In all three genotypes, the overall abundance of Astn2 transcripts are similar and isoforms with and without exon 4 are present. Astn2 transcripts in the Fz6 -/- ;ridge/ridge sample are missing exon 5. (C) Kyte-Doolittle hydropathy profile for Astn2. The locations of the predicted signal peptide and two trans-membrane segments are indicated. (D) Predicted transmembrane topography for Astn2 showing the locations of regions coded by exons 4 and 5, and regions with homology to known domains. FN III, fibronectin type III domain; MACPF, membrane attack complex/perforin domain. (TIF) [file pgen.1005532.s006.tif]

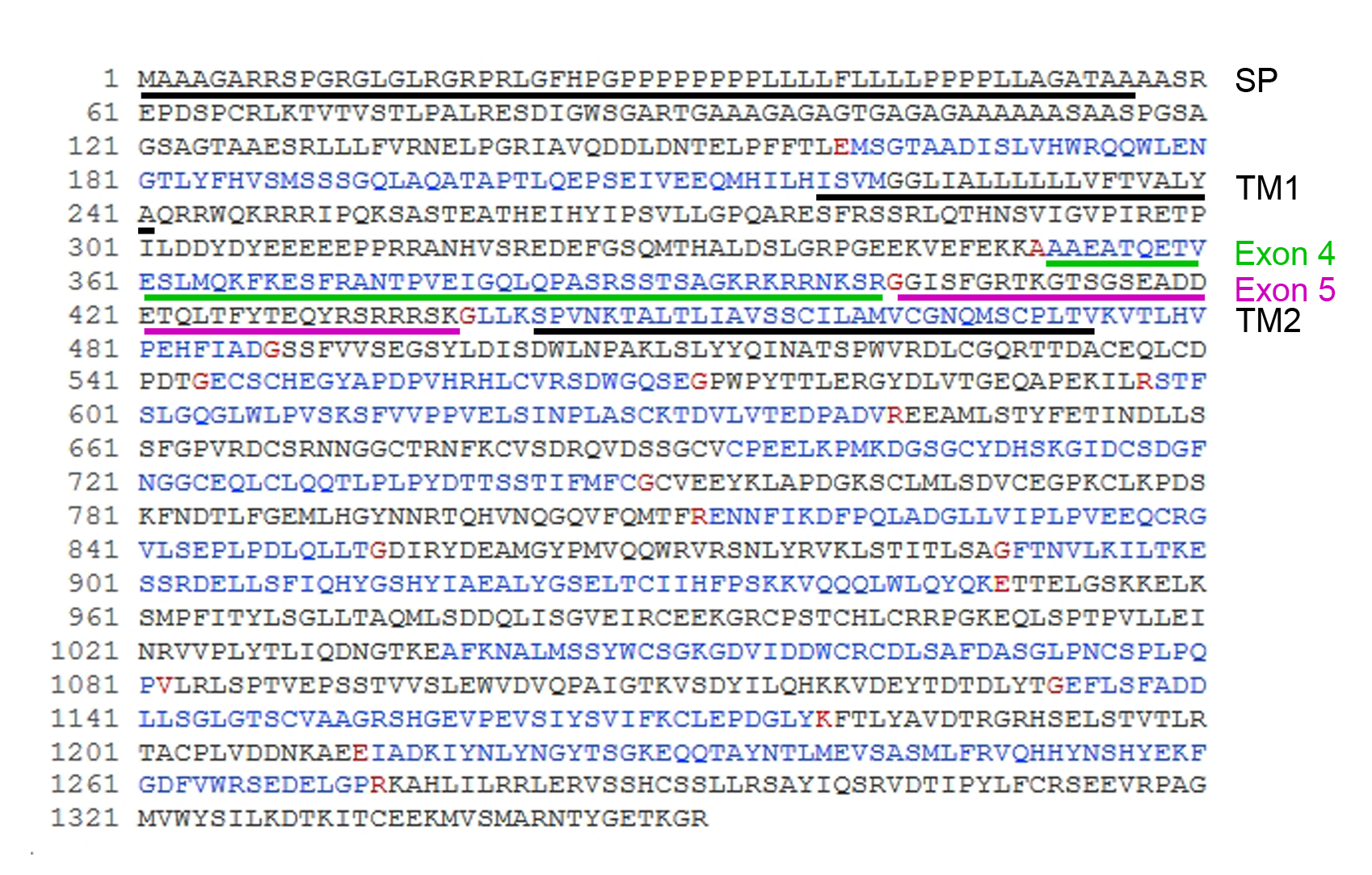

Supplement: S7 Fig — Green underline, exon 4. Purple underline, exon 5. Alternating blocks of black and blue letters represent amino acids coded within different exons. Red letters indicate locations where an intron falls within a codon. The predicted locations of the signal peptide (SP) and the two transmembrane domains (TM1 and TM2) are indicated. (TIF) [file pgen.1005532.s007.tif]

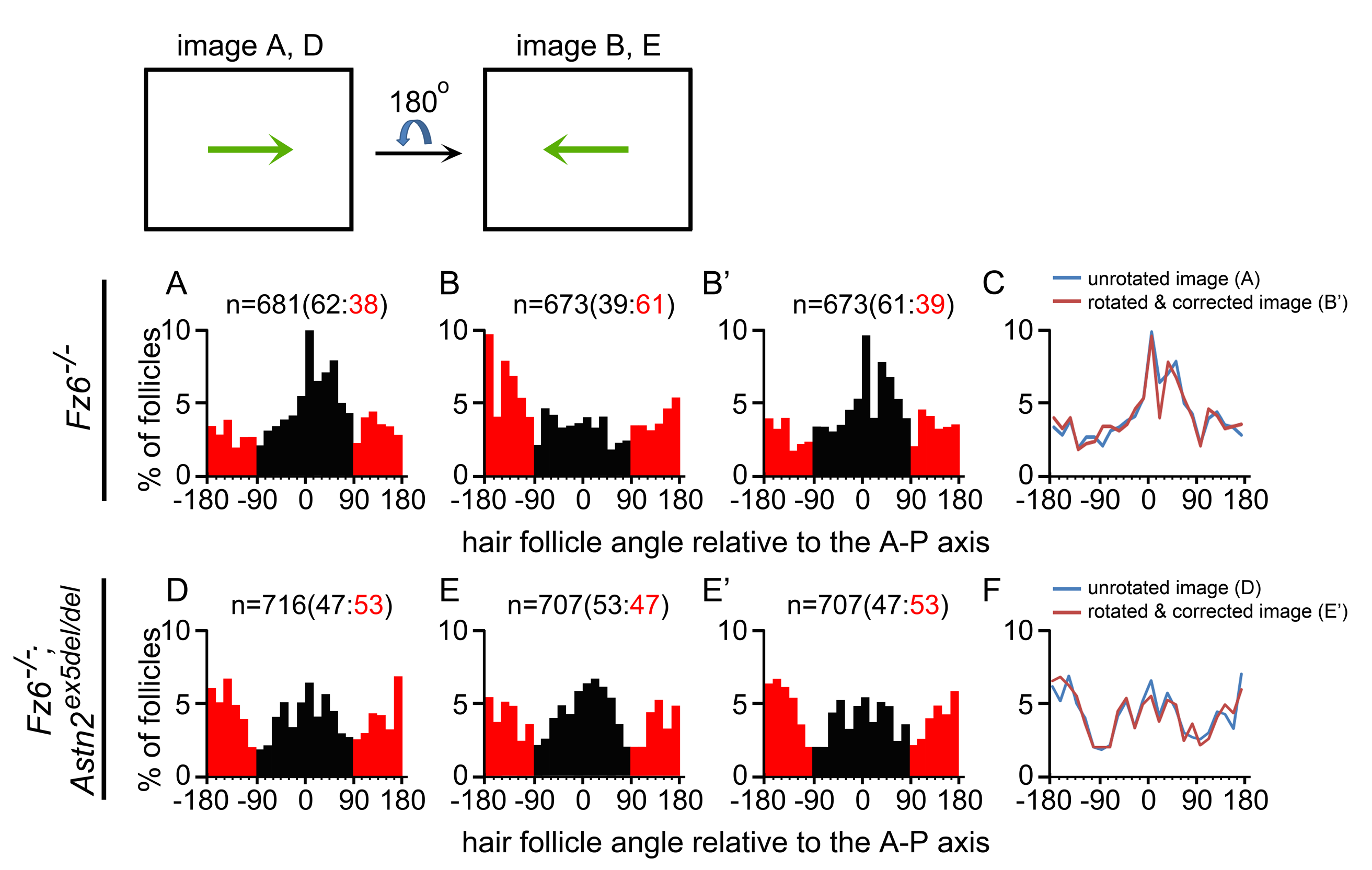

Supplement: S8 Fig — Images of two P3 back skin flat mounts (one Fz6 -/- and the other Fz6 -/- ;Astn2 ex5del/del) were rotated 180 degrees, and the orientations of all follicles within the four images (two original and two rotated) were determined as described in Methods. The scorer was blinded to the genotypes and to the relatedness of the images. A, B, D, E, Follicle orientation histograms are shown for the two original (A and D) and two rotated (B and E) images. For each image, the number of follicles scored and the ratio of left-to-right (black) and right-to-left (red) vectors is shown. In the original images, anterior was to the left and posterior was to the right. B’ and E’, Histograms of the rotated data set after correction for the 180-degree rotation. C and F, Distributions of follicle angles for the original images (blue lines) and the two rotated images after correction for the 180-degree rotation (red lines). (TIF) [file pgen.1005532.s008.tif]
